# Isolation of a Seawater Tolerant *Leptospira* spp. from a Southern Right Whale (*Eubalaena australis*)

**DOI:** 10.1371/journal.pone.0144974

**Published:** 2015-12-29

**Authors:** Sylvia Grune Loffler, Virginia Rago, Mara Martínez, Marcela Uhart, Monica Florin-Christensen, Graciela Romero, Bibiana Brihuega

**Affiliations:** 1 Institute of Pathobiology, National Institute of Agricultural Technology, Hurlingham, Buenos Aires, Argentina; 2 National Research Council of Argentina (CONICET), Buenos Aires, Argentina; 3 Institute of Ecology, Genetics and Evolution, National Research Council of Argentina (CONICET), University of Buenos Aires, Buenos Aires, Argentina; 4 Southern Right Whale Health Monitoring Program, Puerto Madryn, Chubut, Argentina; 5 One Health Institute, School of Veterinary Medicine, University of California Davis, Davis, California, United States of America; University of Kentucky College of Medicine, UNITED STATES

## Abstract

Leptospirosis is the most widespread zoonotic disease in the world. It is caused by pathogenic spirochetes of the genus *Leptospira* spp. and is maintained in nature through chronic renal infection of carrier animals. Rodents and other small mammals are the main reservoirs. Information on leptospirosis in marine mammals is scarce; however, cases of leptospirosis have been documented in pinniped populations from the Pacific coast of North America from southern California to British Columbia. We report the isolation of a *Leptospira* spp. strain, here named Manara, from a kidney sample obtained from a Southern Right Whale (*Eubalaena australis*) calf, which stranded dead in Playa Manara, Península Valdés, Argentina. This strain showed motility and morphology typical of the genus *Leptospira* spp. under dark-field microscopy; and grew in Ellinghausen-McCullough-Johnson-Harris (EMJH) medium and Fletcher medium after 90 days of incubation at 28°C. Considering the source of this bacterium, we tested its ability to grow in Fletcher medium diluted with seawater at different percentages (1%, 3%, 5%, 7% and 10% v/v). Bacterial growth was detected 48 h after inoculation of Fletcher medium supplemented with 5% sea water, demonstrating the halophilic nature of the strain Manara. Phylogenetic analysis of 16S rRNA gene sequences placed this novel strain within the radiation of the pathogenic species of the genus *Leptospira* spp., with sequence similarities within the range 97–100%, and closely related to *L*. *interrogans*. Two different PCR protocols targeting genus-specific pathogenic genes (G1-G2, B64I-B64II and LigB) gave positive results, which indicates that the strain Manara is likely pathogenic. Further studies are needed to confirm this possibility as well as determine its serogroup. These results could modify our understanding of the epidemiology of this zoonosis. Until now, the resistance and ability to grow in seawater for long periods of time had been proven for the strain Muggia of *L*. *biflexa*, a saprophytic species. To the best of our knowledge, this is the first isolation of a *Leptospira* sp. from cetaceans. Our phenotypic data indicate that strain Manara represents a novel species of the genus *Leptospira*, for which the name *Leptospira brihuegai* sp. nov. is proposed.

## Introduction

Leptospirosis is a neglected zoonotic disease, endemic in most tropical and subtropical regions of the world. The causative agents of this zoonosis are pathogenic strains belonging to the order Spirochaetales, family Leptospiracea and genus *Leptospira* spp. Leptospirosis is maintained in nature through chronic renal infection of carrier animals, with rodents and other small mammals as the most important reservoirs [1–3].

In marine animals, seropositivity has been reported in North America, in California sea lions (*Zalophus californianus*) [4], Northern fur seals (*Callorhinus ursinus*) [5], Northern elephant seals (*Mirounga angustirostiris*) [6], and harbor seals (*Phoca vitulina*) [7,8]. Furthermore, *Leptospira* spp. can cause disease in pinnipeds [9–12], periodic large scale stranding and mortality events, every three to four years, of California sea lions along the pacific coast of North America from southern California to British Columbia have been attributed to *L*. *interrogans* serovar Pomona infections [4, 10, 11, 12, 13,14]. Seroprevalence in marine mammals has been reported also in the Pacific coast of South America, in Peruvian Amazon manatees (*Trichechus inunguis*) [15] and in Chilean South American sea lions (*Otaria byronia*) [16].

Cameron et al. [10], describe clinical signs of Leptospirosis in sea lions as renal failure, dehydration, polydipsia, vomiting, and depression. Using species-specific primer pairs Cameron et al. [10] revealed host specificity of *L*. *interrogans* for sea lions and of *L*. *kirschneri* for elephant seals.

The genus *Leptospira* spp. displays a great genomic plasticity and several schemes for the genotyping of pathogenic species have been developed during the last years around the world [17–19]. Bacterial isolation followed by molecular characterization has recently been successfully applied to genotype pathogenic *Leptospira* spp. strains in Argentina. A considerable number of pathogenic *Leptospira* spp. from wildlife [20–22], domestic animals [23–25] and water samples [26] were thus characterized.

Environmental factors are key determinants in leptospiral distribution, as water is the vehicle by which leptospires travel and disseminate into ecosystems. To date, only one strain of *Leptospira* spp., *L*. *biflexa* strain Muggia, has been isolated from seawater in a region near Trieste, Italy [27, 28]. Growth of other *Leptospira* spp., including *L*. *interrogans*, in the presence of diluted seawater has always yielded negative results [2, 3, 29, 30]. However, Saito et al. [31] isolated two leptospiral strains after a typhoon in Philippines in soil samples, and commented that both isolated strains could live in seawater for only three and four days respectively.

The present work describes the characterization of a *Leptospira* sp. strain, isolated from the kidney of a stranded Southern Right Whale in Patagonia, Argentina, that grows optimally in the presence of seawater. This is the second halophilic *Leptospira* spp. reported to date, and to the best of our knowledge, the first one ever isolated from a cetacean.

## Materials and Methods

### Sample collection

A total of 27 kidney samples from 27 dead stranded Southern Right Whales (*Eubalaena australis*) Península Valdés, Argentina, were collected by the Southern Right Whale Health Monitoring Program (SRWHMP) between 2009 and 2010. Field permits for this work were issued annually by the Department of Wildlife of the Subsecretary of Tourism and Protected Areas, Chubut Province, Argentina. Necropsies were performed using a Right Whale necropsy protocol developed by the SRWHMP (Chirife et al., 2014 unpublished) based on the methods of McCellan et al. [32], F. Gulland (pers. comm.), A. Carribero (unpublished) and Geraci & Lounsbury [33]. Carcass decomposition was graded subjectively on a scale from condition code 2 to 5 (2 = fresh, 3 = decomposed but tissues largely intact, 4 = advanced decomposition, 5 = mummified or skeletonized) [33]. Samples were placed in Whirlpack® bags, stored in liquid nitrogen at the site, and later transferred on dry ice to the laboratory for diagnosis. Samples were stored at -70°C until processing.

### Isolation and maintenance of cultures

All kidney samples were cultured in leptospire-specific Ellinghausen-McCullough-Johnson-Harris (EMJH) and Fletcher media (Difco Laboratories). Cultures were incubated at 28°C for 90 days and observed every 15 days under dark-filed microscopy to evaluate possible bacterial growth. Once the culture was positive, subcultures were conducted to maintain the isolated strain alive.

#### Growth assay in the presence of seawater

After weak growth in both EMJH and Fletcher media and considering the source of isolation, we tested the ability of these bacteria to grow in each of these media after the addition of seawater at different percentages (1%, 3%, 5%, 7% and 10%, v/v) [30,31]. Seawater was collected from Puerto Madryn, Chubut, Argentina, and sterilized by filtration through 0.22 μm Millipore filters. Cultures were incubated at 28°C for up to 12 days. A control experiment was carried out, where filter-sterilized seawater was cultured with both EMJH and Fletcher Media. Immunofluorescence staining of the strain was performed with a multivalent FA, LEP-FAC (Seasinglab, Argentina) conjugate specific for *Leptospira* spp.

### Molecular characterization

Two different PCR protocols were tested to indicate if the isolated strain was pathogenic, amplifying genus-specific genes that are considered pathogenicity determinants. DNA templates were obtained after nucleic acid purification using the Chelex-100 resin (Bio Rad) protocol [22].

#### Multiplex PCR

The combined primers G1-G2 and B64I-B64II previously described in Gravekamp et al., [34] were used in this study. The PCR reaction was carried out in a final volume of 50 μl, which contained 2 μl purified DNA template, and the PCR mixture and the cycling program used was the same as indicated in Gravekamp et al. [34]. PCR was carried out in a My Cycler ^TM^ thermocycler (Bio Rad). Amplification products were analyzed by electrophoresis in ethidium bromide stained 2% agarose gels, followed by exposure to UV light (Uvi Tec transiluminator BTS-20.M). Amplicon sizes were estimated using a 100 bp ladder (Embiotech).

#### PCR for LigB

A 1 kb sequence of the adhesin Lig B gene, which is present in all pathogenic *Leptospira* spp. strains, was amplified by PCR using primers LigBpetF and LigBpetR [35]. The PCR mixture and cycling was carried out as indicated in Martínez et al. [35]. PCR was carried out in a My Cycler TM thermocycler (BIO RAD) and amplified samples were analyzed by electrophoresis in 1% agarose gels stained with Sybr® Safe (Invitrogen) and visualized under UV light.

#### 16S rRNA sequencing

A PCR targeting the 16S rRNA gene was carried out for bacterial identification after sequencing. The following primers were used: 5′-GGCGGCGCGTCTTAAACATG-3′ and 5′-GTCCGCCTACGCACCCTTTACG-3′ [36]. These primers have the ability to amplify all pathogenic and nonpathogenic species of *Leptospira* spp. PCR was performed as indicated in Djadid et al. [36]. After verification of the amplicon by electrophoresis in an ethidium bromide-containing 2% agarose gel and visualization upon UV light exposure, PCR products were purified using a commercial kit (EMBIOTECH). The samples were sequenced at the Institute of Biotechnology, National Institute of Agricultural Technology using a 3130xl Genetic Analyzer (Applied Biosystems). For alignment and construction of the phylogeny, the program MEGA version 6.06 [37] was used. The dendrogram was constructed using Neighbor-joining with a bootstrap of 100, partial sequences of the 16S rRNA gene were used.

## Results and Discussion

### Isolation of *Leptospira* sp. and maintenance of the cultures

One positive culture of *Leptospira* sp. was obtained from one Southern Right Whale, a newborn female calf (ID 092410PV-Ea23), 5.05 meters length. The calf stranded dead on September 24^th^ 2010 at Playa Manara in Golfo Nuevo, Península Valdés (42° 40’ 18.9”S 64° 59’ 22.9”W), and was necropsied the following day. Carcass condition at necropsy was 3.

**Fig 1:**
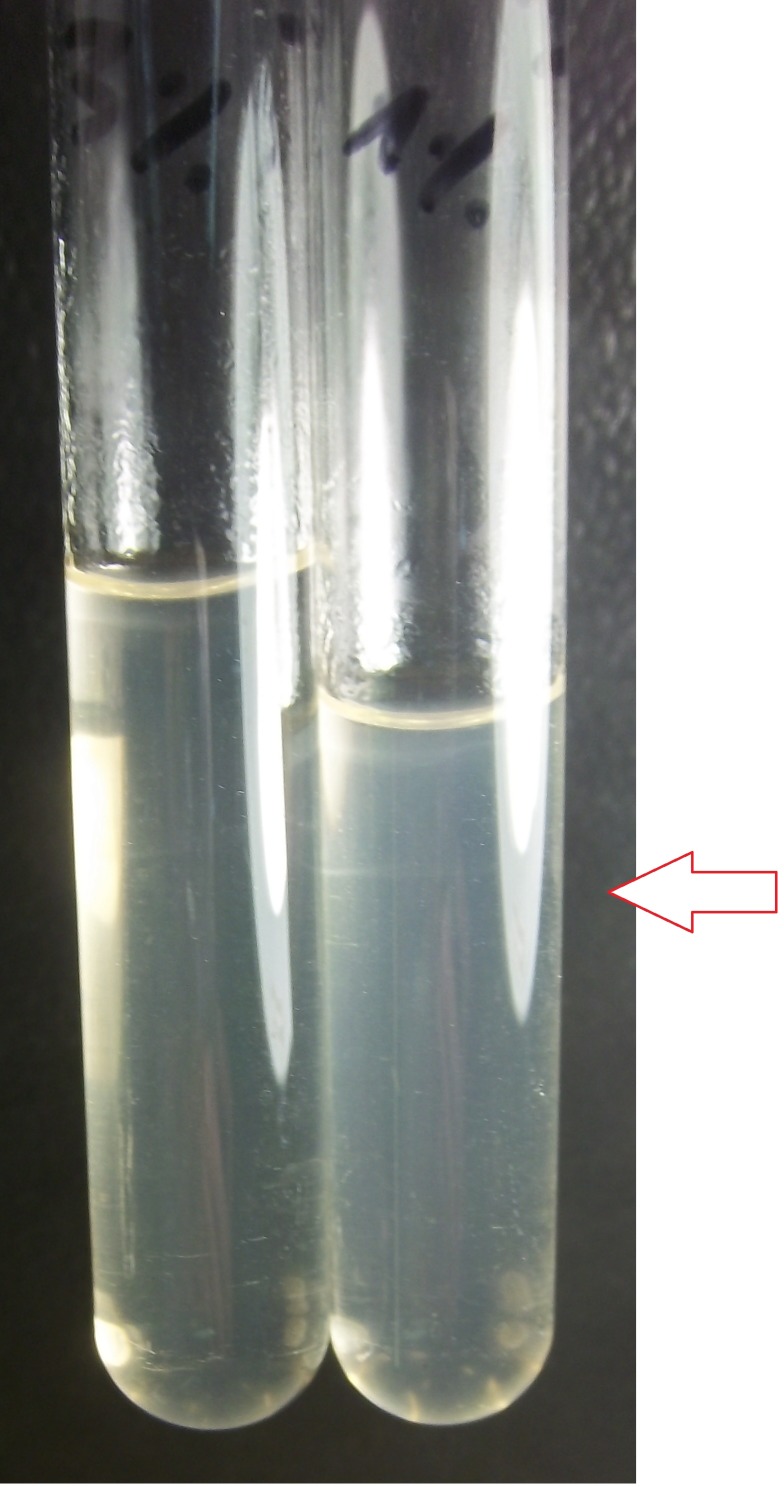
Picture of two cultures of *Leptospira* sp. strain Manara. Two cultures of *Leptospira* sp. strain Manara in Fletcher medium supplemented with 3% (left tube) and 1% (right tube) seawater after 48 h of incubation at 28°C. The dinger rings corresponding to *Leptospira* sp. growth are indicated with red arrows.

The isolated strain was designated strain Manara. This strain showed typical motility and morphology of the genus *Leptospira* spp. under dark-field microscopy and grew in EMJH and Fletcher media after 90 days of incubation at 28°C. When seawater (1%, 3%, 5%, 7%, 9% and 10%, v/v) was added, no growth was observed in EMJH medium during the first 6 days. At day 7, positive growth based on the turbidity of the culture was detected at 1% seawater (Fig 1). A control experiment, where only seawater was incubated in the same media did not show any leptospiral growth. On the other hand, growth of strain Manara was observed at 48 h in Fletcher medium, with a typical dinger ring formation in the culture. After 72 h, growth was achieved at percentages of 1% and 3%, and after 96 h, all cultures were positive (Table 1). At day 8 of this assay, new subcultures were started in Fletcher medium supplemented with the same seawater dilutions as before (Table 2). Importantly, the same growth behavior was observed. These experiments confirmed the ability of strain Manara to grow *in vitro* in the presence of diluted seawater demonstrating its halophilic nature.

**Table 1:** Growth results of strain Manara in EMJH and Fletcher media diluted with seawater.

	EMJH Medium					Fletcher Medium				
day	1%	3%	5%	7%	10%	1%	3%	5%	7%	10%
1	**-**	**-**	**-**	**-**	**-**	**-**	**-**	**-**	**-**	**-**
2	**-**	**-**	**-**	**-**	**-**	**-**	**-**	**+**	**-**	**-**
3	**-**	**-**	**-**	**-**	**-**	**+**	**+**	**+**	**-**	**-**
4	**-**	**-**	**-**	**-**	**-**	**+**	**+**	**+**	**+**	**+**
5	**-**	**-**	**-**	**-**	**-**	**+**	**+**	**+**	**+**	**+**
6	**-**	**-**	**-**	**-**	**-**	**+**	**+**	**+**	**+**	**+**
7	**+**	**-**	**-**	**-**	**-**	**+**	**+**	**+**	**+**	**+**
8	**+**	**-**	**-**	**-**	**-**	+*	+*	+*	+*	+*
9	**+**	**-**	**-**	**-**	**-**	**+**	**+**	**+**	**+**	**+**
10	**+**	**-**	**-**	**-**	**-**	**+**	**+**	**+**	**+**	**+**
11	**+**	**-**	**-**	**-**	**-**	**+**	**+**	**+**	**+**	**+**
12	**+**	**-**	**-**	**-**	**-**	**+**	**+**	**+**	**+**	**+**

Growth results of strain Manara in EMJH and Fletcher media diluted with seawater at percentages 1%, 3%, 5%, 7% and 10% (v/v). + and–correspond to detection and no detection of growth, respectively; Subcultures (marked with an asterisk) under the same conditions were started at day 8.

**Table 2:** Growth results of subcultures of strain Manara in Fletcher media diluted with seawater after 8 days.

	Subculture from Fletcher Medium after 8 days				
day	1%	3%	5%	7%	10%
1	-	-	-	-	-
2	-	-	-	-	-
3	+	+	+	+	+
4	+	+	+	+	+
5	+	+	+	+	+

Growth results of subcultures strain Manara started at day 8 of the first culture assay, in Fletcher media diluted with seawater at percentages 1%, 3%, 5%, 7% and 10% (v/v). + and–correspond to detection and no detection of growth, respectively.

**Fig 2:**
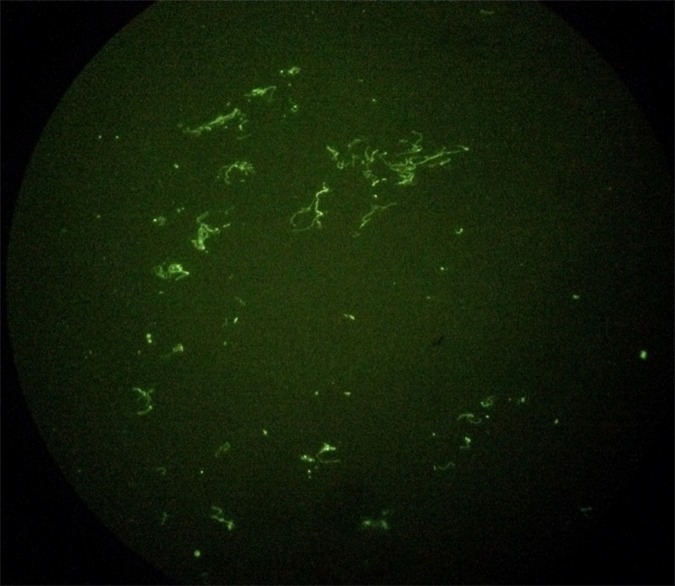
Immunofluorescence of strain Manara. Immunofluorescence of strain Manara isolated from a kidney sample of a Southern right whale (*Eubalaena australis*) 40X.

Immunofluorescence detection was positive and leptospires could be microscopically observed. (Fig 2).

To the best of our knowledge, there are no recognized halophilic pathogenic leptospiral strains. Previous studies have demonstrated that *Leptospira* spp. can grow in seawater [27, 28] but the identification of serogroup and serovar was not provided and the authors indicated that this unique strain Muggia could belong to the species *L*. *biflexa*. In the study of Cinco et al. [28], the isolated halophilic strain grew in Korthof-Babudieri seawater medium for about 16 to 18 h, and a maximal concentration of 2 x 10^8^ cells/ml was reached after 10 days of incubation at 30°C. In their study, the strain was considered as halodependent to specific NaCl requirements for growth. On the other hand, Saito et al. [31], could isolate two leptospiral strains from soil samples and tested the ability to grow in seawater, one strain called MS422 could grew in seawater for four days and the second strain MS432 only for three days. Both strains grew also in media with PBS for six days. These studies did not extend for a longer time period. In the same study [31], both strains were sequenced using the 16S rRNA gene and both showed similarity to *L*. *kmetyi*.

In our study, the strain Manara was capable of growing in the presence of different concentrations of seawater (Table 1). In media that was not supplemented with seawater, growth of Manara strain was considerably slower and could not be detected until 30 days (results not shown), whereas growth could be detected as soon as 48 h after inoculation when cultured in media with 5% seawater in Fletcher media. Further studies are necessary to characterize the biological features of this novel strain.

### Molecular characterization

**Fig 3:**
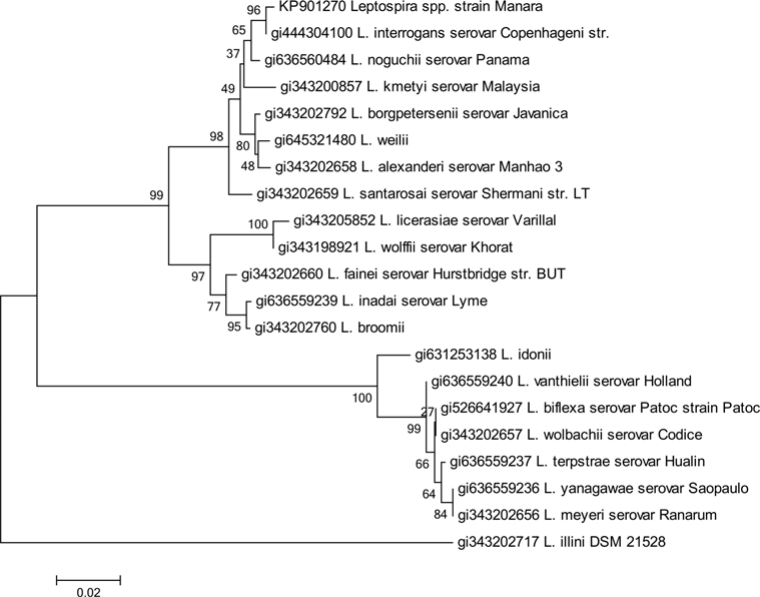
Neighbor-joining analysis of the sequence 16S rRNA of strain Manara. Phylogenetic analysis based of 16S rRNA including 19 representative species of *Leptospira* spp. The corresponding sequence of *Leptonema illini* strain DSM 21528 was used as outgroup. The dendrogram was constructed using Neighbor-joining. Bootstrap values are displayed as percentages.

Two different PCR protocols were used to explore the possible pathogenicity of the Manara strain, amplifying the genus-specific and virulence-associated genes G1-G2, B64I-B64II and LigB, both with positive results. Phylogenetic analysis of 16S rRNA gene sequences placed this novel strain within the radiation of the pathogenic species of the genus *Leptospira* spp., with sequence similarities within the range 97–100% (results not shown), and closely related to *L*. *interrogans* (Fig 3). Further molecular characterization must be pursued to confirm this possibly new species, such as sequencing secY gene. The sequence obtained is available in GenBank under Accession number KP901270.

Detection of new strains in the environment and carrier animals is vital to the understanding of enzootic and epizootic leptospirosis in marine mammals. Since pathogenic *Leptospira* spp. serovars are extremely difficult to cultivate *in vitro* [29,30], the mode of transmission of this organism in marine species is not understood [10–13]. Detection by PCR of leptospiral DNA in sand was observed by Cameron et al. [10], however this study could not determine if *Leptospira* sp. was dead or alive in sand. In this study, no samples were taken at site, where the Southern Right Whale stranded dead, but the possibility exists that leptospiral DNA could be also in sand. Potential environmental sources of pathogen exposure in the marine environment could increase the zoonotic potential of Leptospirosis in marine mammals, however the way of transmission is yet unclear [5,7,8,10,11,12,14].

Confirmation that this isolate contained the gene LigB, a gene associated with virulence and present in all pathogenic *Leptospira* spp., suggests this strain is pathogenic and is consistent with our sequencing results which place strain Manara within the radiation of other pathogenic *Leptospira* spp. Further studies in animal models must be conducted to confirm this possibility. In addition, determination of the serogroup and serovar of this strain are the object of on-going research.

Due to the phenotypic findings in this study we propose that strain Manara belongs to a new species of *Leptospira* spp., phylogenetically closely related to *L*. *interrogans*, but with the particular features of being able to survive during a long period of time in seawater, and to colonize unreported hosts, as the Southern right whale.

#### Ethiology of *Leptospira brihuegai* sp. Nov

The proposed name for strain Manara is *Leptospira brihuegai*, after professor Bibiana Brihuega, an Argentinean veterinarian and bacteriologist who has made significant contributions to the study of Leptospirosis.

## Conclusion

This study reports the first isolation of a *Leptospira* sp. from a cetacean. Phylogenetic analysis of a sequence obtained by targeting the 16S rRNA as well as its host specificity and growth requirements identified this as a novel halophilic *Leptospira* sp. strain These findings contribute highly relevant information to current knowledge on leptospirosis epidemiology and ecology

New findings concerning the biology of leptospires is a challenge and more extensive studies are required to monitor the presence of *Leptospira* spp. in the environment and different mammalian hosts. Our study contributes valuable information to our knowledge on leptospires overall, and on marine mammals in particular.
